# Poisoning by Belladonna

**Published:** 1869-09-15

**Authors:** N. A. Drake

**Affiliations:** Ossian, Iowa


### *Poisoning by Belladonna.*

OSSIAN, IOWA, Aug. 2, 1869.

EDITOR CHICAGO MEDICAL JOURNAL:

The following case may be of interest as it demonstrates, to me at least, the antagonism between belladonna and opium.

Was called in great haste to see a woman a mile out of town. I got there as soon as possible and found the old lady perfectly delirious. Face swollen and flushed; eyes injected and pupils dilated to their fullest extent; would, stagger and fall when she attempted to walk; tongue swollen and could not speak audibly; pulse small and flickering; was constantly reaching after imaginary objects in the air.

My diagnosis was, poison by belladonna.

I inquired if she had been taking anything, when one of the children said that Dr. Small had been there. This Small is a drunken traveling quack, who is stopping awhile in our town.

I got them to give me the medicine, which made me sure in my diagnosis. She had vomited just before I got there, and had taken the medicine, as near as I could learn, about four hours before. I was preparing to give an emetic when she vomited again. I then gave her one half grain of acetate of morphia, and repeated it every half hour till the pupil began to contract, which it did after the third dose. I then lessened the dose, and continued at longer intervals, till reason was fully restored, which was about fifteen hours after she took the first dose of morphia.

I called at the drug store and requested to see the prescription, having the medicine with me. And here it is, and you can see that one dose contains about fifteen grains of Ex. Belladonna.

℞. Muriate Ammonia ℥iv.

Ex. Belladonna ℥ij.

Aq. font ℥xij.

Mx.

Dose—ij℥ twice a day.

I am of the opinion if the old lady had not vomited freely it would have been all day with her.

Yet I am confident, from watching her closely, that morphine, in this case at least, produced a decidedly antagonistic effect. Yours,

N. A. Drake, M.D.

